# The Importance of Early Genetic Diagnostics of Hearing Loss in Children

**DOI:** 10.3390/medicina56090471

**Published:** 2020-09-14

**Authors:** Nina Božanić Urbančič, Saba Battelino, Tine Tesovnik, Katarina Trebušak Podkrajšek

**Affiliations:** 1 Department of Otorhinolaryngology and Cervicofacial Surgery, University Medical Centre Ljubljana, Zaloska 2, 1000 Ljubljana, Slovenia; saba.battelino@kclj.si; 2 Department of Otorhinolaryngology, Faculty of Medicine, University of Ljubljana, Vrazov trg 2, 1000 Ljubljana, Slovenia; 3 University Children’s Hospital, University Medical Centre Ljubljana, Bohoriceva 20, 1000 Ljubljana, Slovenia; tine.tesovnik@kclj.si (T.T.); katarina.trebusakpodkrajsek@mf.uni-lj.si (K.T.P.); 4 Faculty of Medicine, Institute of Biochemistry, University of Ljubljana, Vrazov trg 2, 1000 Ljubljana, Slovenia

**Keywords:** genetic testing, etiology, benefits, health, family

## Abstract

Hearing loss is one of the most common sensory deficits. It carries severe medical and social consequences, and therefore, universal newborn hearing screening was introduced at the beginning of this century. Affected patients can have hearing loss as a solitary deficit (non-syndromic hearing loss) or have other organs affected as well (syndromic hearing loss). In around 60% of cases, congenital hearing loss has a genetic etiology, where disease-causing variants can change any component of the hearing pathway. Genetic testing is usually performed by sequencing. Sanger sequencing enables analysis of the limited number of genes strictly preselected according to the clinical presentation and the prevalence among the hearing loss patients. In contrast, next-generation sequencing allows broad analysis of the numerous genes related to hearing loss, exome, or the whole genome. Identification of the genetic etiology is possible, and it makes the foundation for the genetic counselling in the family. Furthermore, it enables the identification of the comorbidities that may need a referral for specialty care, allows early treatment, helps with identification of candidates for cochlear implant, appropriate aversive/protective management, and is the foundation for the development of novel therapeutic options.

## 1. Introduction

### 1.1. Hearing Loss

Hearing loss is the most common sensory deficit, as 1 in 1000 neonates have severe to profound, permanent hearing loss [1]. When all degrees of hearing loss are considered, the prevalence significantly increases. By the age of 18 years, 17 in 1000 individuals are affected by various degrees of permanent hearing loss, while such an increase can be attributed to progressive, acquired, or late-onset genetic causes [2].

We can divide hearing loss by the place where it occurs. When it is caused by the malfunctioning of the outer and middle ear (auricle to the cochlea), it is addressed as conductive hearing loss (CHL). On the other hand, when the reason is in the cochlea, auditory nerve, auditory nuclei, or auditory cortex, we use the term sensorineural hearing loss (SNHL). In the mixed hearing loss, both causes are present at the same time.

Regarding the timing of the onset, hearing loss can occur before the acquisition of speech—prelingual, after the acquisition of speech-post lingual, in adulthood- adult-onset or later; age-related onset or presbycusis. Regarding progression, hearing loss can be progressive, nonprogressive, or fluctuating. It can be unilateral or bilateral. The pure tone audiogram curve could have a sloping, flat, rising, or mid-frequency loss (cookie bite) configuration, which is essential in genetic diagnostics of hearing loss [3]. While the sloping curve is more characteristic of acquired hearing loss [4], the cookie bite curve tends to be characteristic of genetic etiology [5]. Patients with mid-frequency loss seldom use hearing aids as their hearing at low and high frequencies is normal.

### 1.2. Universal Newborn Hearing Screening

Unilateral and bilateral hearing loss have severe consequences on a child’s development if left untreated. They might negatively affect language development, classroom learning, academic performance, and social development [6]. Unilateral hearing loss has an impact on audition, school performance, and neurocognitive factors [7]. Because of those severe consequences, universal newborns hearing screening (UNHS) was introduced at the beginning of this century in North America (2001) [3], Europe, and most developed countries [2]. In 2005, all Slovenian birth hospitals began screening newborns during the first three days after birth. The main goal of UNHS is to identify children with all types and degrees of hearing impairment and to lower the age when the diagnosis is set. This would consequently enable early hearing amplification and maximization of their linguistic competence and literacy development [8]. Although UNHS became an essential tool in the early detection of newborn children with hearing loss, unfortunately, it has some pitfalls. Namely, a standard screening test (transitory evoked otoacoustic emissions—TOAE) evaluates only the function of outer hair cells [9], leaving all the other structures (inner hair cells, the nerves, central structures, etc.) untested. This is the reason why it cannot identify children with auditory neuropathy (malfunctioning of the auditory nerve). The other approach that is less frequently used, at this moment in Slovenia only for newborns hospitalized in the newborn intensive care unit, is auditory brainstem response (ABR) or a less time-consuming simplified version—automated auditory brainstem response (aABR). This test enables us to make some conclusions about central auditory processing and detect children with pathological processes affecting this part of the hearing pathway. The other concern regarding UNHS is that it does not identify children with progressive hearing loss, namely children that develop hearing loss later during childhood and adolescence. Unfortunately, standardized hearing screening for children before school entry is not established in Slovenia, even though it is very much needed. Children at that age are at the very beginning of the intense period of their intellectual and social growth and hearing importantly supports full use of their potentials. Both shortcomings of the current UNHS approaches could be overcome by early genetic screening for hearing loss.

## 2. Genetic Etiology of Hearing Loss

The fact that the majority of newborns have a hereditary cause for their hearing loss is being obscured by the fact that ninety-five percent of newborns with hearing loss identified by newborn hearing screening programs are born to hearing parents [10].

The majority of congenital hearing loss, up to 60% of cases, is due to a genetic etiology [2]. Hearing loss-causing gene variants can affect any component of the hearing pathway [2], for example, genes encoding different proteins that affect the development and function of the ear, transcription factors, structural proteins, ion channels proteins, gap junction proteins, and so on [11]. We can define the genetic phenotypes of sensorineural hearing loss (SNHL) as syndromic or non-syndromic. Non-syndromic SNHL (NSSNHL) accounts for the majority (70%) of congenital hearing loss cases in developed countries [12]. There are over 120 non-syndromic hearing loss genes identified to date [13], since initial gene identification in 1995 [14], and there are many additional loci identified associated with hearing loss, although the genes are unknown. In total, 30% of congenital hearing loss cases are syndromic and occur together with structural or functional anomalies of other organs and systems. In addition, age-related hearing impairment is believed to be associated with the cumulative effect of environmental and genetic factors. In this context, genetic factors are poorly understood, although large genome-wide association studies (GWAS) identified several novel disease-associated loci [15] and massive next-generation sequencing might bring additional knowledge in this field.

### 2.1. Non-Syndromic Hearing Loss

Numerous genes are related with non-syndromic hearing loss that can be transmitted in an autosomal recessive, autosomal dominant, x-linked, or mitochondrial pattern of inheritance [13].

Autosomal-recessive (AR) inheritance accounts for 80% of non-syndromic genetic hearing loss, predominantly being prelingual [16]. Autosomal-dominant (AD) inheritance accounts for most of the other 20% and is contrary to the recessive form, more often post lingual [16]. AR non-syndromic hearing loss most frequently results in severe hearing loss, which presents early [16]. In contrast, AD non-syndromic hearing loss typically results in progressive sensorineural hearing loss (SNHL) that has variable severity and onset between the ages 10 and 40 years [17]. Patients with mitochondrial inheritance predominately have progressive SNHL of variable severity, with onset between the ages of 5 and 50 years [18]. X-linked and mitochondrial inheritance accounts for only 1% to 2% of non-syndromic hearing loss [16].

In the case of AR inheritance, there are usually no other family members with the same type of hearing loss. Most of the patients with non-syndromic SNHL have the disease-causing variant in the gene *GJB2*, which encodes the protein connexin 26 [19], a gap junction protein, an important component of the intracellular pathway for potassium cycling between the endolymph and perilymph of the cochlea [16]. Hearing loss due to *GJB2* deficiency was first described in 1997, and since then, worldwide *GJB2* coding region sequencing has demonstrated various spectrums of genotypes across different populations. A particularly prevalent *GJB2* disease-causing variant is a deletion NM_004004.6: c.35del (p.Gly12fs) with a carrier frequency 1/35 in southern and 1/79 in central and northern Europe [20] and 1/72 in the Middle East [21]. In the Slovenian population, disease-causing variants in the *GJB2* gene are present in 26.6% of patients with congenital hearing loss [22] and 22.2% of patients with progressive hearing loss [23]. While large deletions of the *GJB6* gene are the second most common reason for prelingual SNHL in some populations (like Spanish) [24], no such deletions were detected in the cohort of 210 congenitally or progressively deaf or hearing impaired Slovenians [25].

Causative variants in the *TMPRSS3* gene, encoding a transmembrane protein that belongs to the serine protease family, are the cause for post lingual or congenital hearing loss [26]. As much as 13% of hard of hearing patients in a carefully preselected Slovenian cohort with suspected autosomal recessive inheritance and no disease-causing variants in *GJB2* or *GJB6* genes had disease-causing variants in the *TMPRSS3* gene [27].

The *TECTA* gene is linked to AD non-syndromic hearing loss and disease-causing variants in the *TECTA* gene are the most frequent cause of dominant hearing loss [28]. It encodes the protein α-tectorin that is a tectorial membrane component [29]. It is associated with mild to profound pre or post lingual hearing loss that could also be of a progressive type and is sometimes connected to cookie bite-type pure tone audiogram [30].

Hearing disorders with mitochondrial inheritance patterns frequently result in a syndromic presentation. The exceptions are causative variants in the mitochondrial 12s ribosomal RNA (MTRNR1) [31] associated with predisposition to aminoglycoside ototoxicity. Individuals who are exposed to aminoglycoside antibiotics and are carriers of MT-RNR1 susceptibility variants develop bilateral, severe to profound hearing loss, typically within a few days to weeks after administration of any amount of the drug [32].

X-linked non-syndromic hearing loss, however, is commonly caused by *POU3F4* variants. A defective *POU3F4* gene is clinically characterized by cochlear hypoplasia, bulbous internal auditory canals, and sensorineural hearing loss with a variable conductive component [33].

In some cases, different disease-causing variants in the same gene can be associated with hearing loss inherited in recessive or dominant mode. An example of such a gene is *MYO7A*, associated with the abovementioned Usher syndrome and rarely to hearing loss, with various severity and autosomal recessive (DFNB2) or autosomal dominant (DFNA11) inheritance [34]. The clinical severity of the disease depends on the associated genetic variant, its location and consequence on the protein level, and consequently, function of the protein. This is particularly evident in proteins with alternative splicing and various functions such as myosin 7A [35].

### 2.2. Syndromic Hearing Loss

Hearing loss is among the most etiologically heterogeneous disorders, with more than 400 genetic syndromes that include hearing loss as a feature [3]. Syndromic hearing loss may be transmitted as an autosomal recessive, autosomal dominant, X-linked, or matrilineal trait. There are more than 40 known genes connected to syndromic hearing loss [13].

In patients with suspected syndromic hearing loss, it is often even more important to determine the genetic cause, as many of the accompanying clinical features can be more severe. Early diagnosis can predict the progression of hearing loss, guide future treatments, and provide a warning for potentially life-threatening abnormalities [36]. For instance, identifying a causative variant in the *SLC26A4* gene (encoding the anion transporter protein named pendrin, related to Pendred syndrome) may be very important for the child’s future. It may help to predict whether the child will develop thyroid dysfunction or goiter after puberty, or might be at risk for hearing loss after head trauma due to an enlarged vestibular aqueduct [37]. Some patients will not develop thyroid problems. They are said to have non-syndromic autosomal recessive hearing loss (locus DFNB4). The estimation is that Pendred syndrome is a cause for 10% of genetic hearing loss [38].

The most common among autosomal recessive syndromic hearing losses is Usher syndrome with the prevalence 1/6000 to 1/25,000 [39]. It is an autosomal recessive disease characterized by deafness and visual impairment due to retinal degeneration (retinitis pigmentosa). Additionally, in some cases, vestibular defects are present [40]. Usher syndrome can be classified into three different types based on clinical findings (type I, II, and III). The majority of patients with Usher syndrome type 2 have causative variants in the *USH2A* gene [41], encoding usherin involved in the function of photoreceptors and cochlear hair cells. The *MYO7A* gene encodes myosin 7A and is causative for 75% of Usher syndrome type I cases [42]. A large study screened a cohort of 427 patients (139 USH1, 282 USH2, and six of undefined clinical subtype) from various European medical centers including Slovenia. It has identified a total of 421 different sequence variants predicted to be pathogenic, about half of which had not been previously reported [43]. Although numerous variants in the *USH2A* gene were reported, as much as 84% of the Slovenian patients with Usher syndrome type 2 had the NM_206933.4:c.11864G>A (p.Trp3955Ter) variant [44]. As blindness due to the retinitis pigmentosa does not occur during infancy but typically at the age of 10 years for Usher type 1, diagnosing Usher syndrome presents its own set of unique challenges. Managing the loss of hearing and vision makes early genetic diagnosis of Usher syndrome necessary [45], as it yields in the appropriate special education training programs.

Jervell and Lange-Nielsen syndrome is the third most common cause of autosomal recessive syndromic hearing loss due to variants in genes encoding for voltage-gated potassium channels [37,46]. The key features are congenital SNHL and prolonged QT interval (greater than 440 ms) [36]. 

Among an autosomal dominant group of syndromic SNHL, the most common is neurofibromatosis type 2 (NF2) with bilateral vestibular schwannomas, meningiomas, and optical gliomas [47]. The incidence is 1 to 25,000 newborns [48]. It is caused by the variants in the neurofibromin 2 (*NF2)* gene encoding the tumor suppression protein [49]. Another autosomal dominant syndromic SNHL is Waardenburg syndrome with variable phenotypic expression, including pigmentation abnormalities of the eyes, hair, skin, and cochlea [50]. Mild to profound hearing loss occurs in 70% to 93% of individuals with Branchio-oto-renal (BOR) syndrome associated with *EYA1* and *SIX5* genes [37]. Other well-known autosomal dominant syndromes with hearing loss are coloboma, heart defects, choanal atresia, retarded growth, genital hypoplasia, and ear abnormalities syndrome (CHARGE) and Treacher Collins syndrome [36].

## 3. Genetic Diagnostics

The conventional sequencing method in genetic diagnostics of any inherited trait is the Sanger dideoxy or enzymatic chain termination method [51]. This approach enables the sequencing of selected short segments of genetic material [52] and is still widely used [51]. However, large-scale sequencing projects, such as the human genome project, boosted the development of novel technologies that are now reducing the required time and costs of the sequencing [51]. In non-syndromic hearing loss, causative variants in several genes result in similar clinical presentation, although causative variants in the same gene can result in numerous clinical presentations [53]. That is the reason why, in most cases of hard of hearing patients, it is almost impossible to choose the appropriate gene for genetic testing with the Sanger approach. Nevertheless, genes such as *GJB2*, commonly mutated in NSHL patients or *SLC26A4*, associated with a particular clinical feature, are still often analyzed with this approach. On the other hand, next-generation sequencing (NGS) enables better sequencing performance with the simultaneous reading of numerous sequences [51]. With this approach, one could simply sequence all or merely selected hearing loss-associated genes with a so-called ‘gene panel’ approach. The analysis could be directed towards non-syndromic or syndromic hearing loss genes or even narrower to the Usher syndrome- or Waardenburg’s syndrome-associated genes [52]. Alternatively, one may choose to sequence only the coding regions of the genome (namely the exome) or even the entire genome, both enabling the identification of novel hearing loss-associated genes [54]. NGS sequencing has high specificity and sensibility, and the diagnostic yield in HL is reported to be between 50 and 60% [54]. This might even be higher when sequencing and interpreting several affected and non-affected family members at the same time. This approach enables easier interpretation of the detected genetic variants, since it allows better discrimination of the variant origin and its pathogenicity. In pediatric patients, trio analysis, including proband and parents, is particularly common. In the study of Slovenian hearing loss patients, after clinical examination and clinical exome sequencing, an etiological diagnosis was established in 30% from the syndromic group and 21% from the non-syndromic non-*GJB2* subgroup [55]. With the panel approach and sequencing of all known deafness-causing genes after *GJB2* has been pre-screened, the diagnostic rate increases to approximately 50% [54].

The result of sequencing is a nucleotide sequence that needs to be compared to the reference sequence of the analyzed gene, such as those collected in the NCBI Genome database [56], which is the resource that organizes information on genomes including sequences, maps, chromosome etc. This enables the identification of the deviation in a sequence and therefore, the location of the gene variant. Interpretation of the identified variants is of utmost importance [57]. It is necessary to carefully evaluate the impact of the identified variant for protein function and consequently, the development of the disorder. In this phase, the use of known databases and literature is crucial [52]. The American College of Medical Genetics published standards and recommendations for the interpretation of the sequence variants [57].

Regarding the known data about the identified sequence variant, the decision regarding its impact on the patient’s clinical picture should be made. Each variant should be categorized as a benign, probably benign, variant of unknown significance, probably pathological, or pathological [57]. Nevertheless, this might be challenging, primarily when we identify novel variants in known disease-causing genes or a gene not entirely associated with the patient’s clinical presentation. It this case the information enabling the final call regarding the pathogenicity of the variant might not be possible and the variant is addressed as a variant of unknown significance (VUS).

### Hearing Loss Diagnostics in the University Medical Centre Ljubljana, Slovenia

In the University Medical Centre Ljubljana, newborns with suspected hearing loss identified by the UNHS program are referred to the Otorhinolaryngology Clinic—Audiovestibulology department outpatient consultation. History taking and a detailed clinical otoneurologic exam (including otomicroscopy) are performed, followed by further hearing testing. As a first step, tympanometry, which helps to define the outer and middle ear function, is performed. Due to the unresponsiveness of TOAE even in mild hearing loss cases, we proceed with TOAE testing only in cases with a good tympanometry result—namely tympanogram type A or eventually C. In cases with no response to TOAE tests, we proceed with audiometry in the free field (with visual reinforcement or play audiometry, depending on a child’s age) using warbled pure tones sounds across the frequencies of 250 to 4000 Hz. Specially trained clinical speech and language therapists perform these tests. In the case of a child’s poor responsiveness, we consider the introduction of a hearing aid. All children with tympanogram A and non-evoked TOAE are objectively tested in induced sleep (with chloralhydrate).

**Figure 1:**
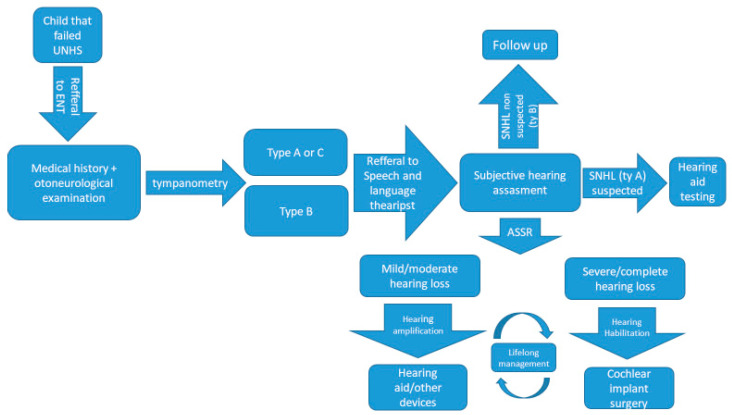
ENT workup of a child with a suspected hearing loss.

**Figure 2:**
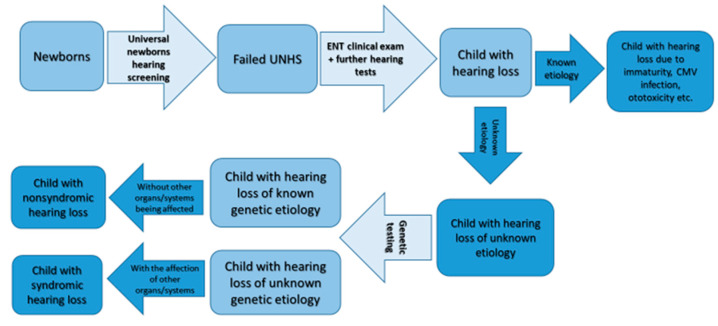
Genetic hearing loss workup.

At the audiovestibulology department of the University Medical Centre Ljubljana, the auditory steady-state response (ASSR) is measured. As a result, we obtain an audiogram-like curve that helps us evaluate the degree of the child’s hearing loss. Some children are referred to the Children’s Hospital neurophysiology laboratory for the measurement of auditory brainstem responses. If permanent hearing loss is identified, an effort is made to provide the child (with the parent’s consent) with a hearing aid by the age of four to six months. If a child is not making progress or has been diagnosed with severe-to-complete hearing loss, cochlear implantation by the age of 11–12 months is planned. All children with suspected or proven hearing loss or children with factors that endanger them for the possible development of hearing impairment have a regular follow up at the Audiolovestibulology centre, where we measure their hearing and speech and language progress and appropriately adjust our interventions. The protocol of patient workup is summarized in Figure 1. If the reason for hearing loss has not been identified, the child and the family are routinely offered genetic testing for the affected patients. If the parents consent to it, the testing is performed at the Clinical institute for Special Laboratory Diagnostics at the University Children’s Hospital, University Clinical Centre Ljubljana where NGS testing for hearing loss was introduced in 2014. At the time, the first step of the testing protocol was Sanger sequencing of the *GJB2* and *GJB6* coding regions followed by targeted NGS sequencing of syndromic and non-syndromic hearing loss-related genes. See the genetic hearing loss algorithm in Figure 2. In individual cases where a certain syndrome is clinically suspected, the analysis is limited to the genes linked to that particular syndrome [52]. Since December 2019 onwards, all patients with suspected hearing loss are directly NGS sequenced. This was enabled by the advances in NGS technology and in-house automatization of the interpretation of the variants. Currently, the analysis targets 124 genes related to syndromic and non-syndromic hearing loss (Table 1) in the patient, followed by segregation analysis in parents. 

## 4. Hearing Loss Therapeutic Options

Hearing is essential for linguistic, social, and intellectual development. Consequently, hearing restoration is a crucial role of audiologists and ENT specialists. In most hard of hearing patients, auditory rehabilitation includes conventional hearing aids. Cochlear implantation (CI) is the surgical insertion of an electrode that provides electrical stimulation directly to the auditory nerve (bypassing the cochlea in which the pathogenetic cause lies). At the present time, CI is the standard therapeutic option for severe-to-profound sensorineural hearing loss patients [53]. From the year 1981, when the first child was implanted with CI [58], the technology has adopted numerous improvements in its function and technical characteristics, aiming to make the sound produced by the implant as natural as it can be. Regardless of all the efforts made in that direction, some problems remain unsolved. Many deaf children are implanted only monaurally (because of the costs and risks of binaural implantation). In addition, the presence of normal anatomical (the cochlea) and neural structures (the auditory nerve) is required for a cochlear implant to attain proper hearing function. Moreover, the use of a CI may be limited by developmental issues and fine and gross motor control (e.g., of the head) [59]. Children should be recognized and treated with a CI before the age of one year, as early stimulation of the auditory pathway is necessary for the development of spoken language [60]. Like all other electronic devices, cochlear implants can malfunction; they should be changed every 15 to 20 years etc. 

Inspired by advances in knowledge regarding the genetic etiology of SNHL and the successes in other specialties, there is a growing interest in gene therapy for hearing loss [61]. It is believed that the time when therapies will have the capability of maintaining or even restoring hearing with more natural sound perception is getting closer, mainly due to increasing knowledge in “repairing” the genes affected by the gene defect [62]. There are two main approaches for gene therapy of inherited hearing loss. The first one is the replacement or augmentation by exogenous expression of wildtype genes and the second one is blocking or eradication of the mutant alleles [62]. In viral transfection of inner ear cells, direct local injection of viral vectors into the inner ear is necessary. Traditional routes for injecting the agents are (1) round window membrane; (2) canalostomy; (3) cochleostomy into either the endolymph or perilymph; and (4) round window membrane combined with canal fenestration [61]. Numerous reports have described gene therapy in neonatal and adult mice models of human hearing loss to be of sporadic success. However, for inner ear gene therapy to enter the clinical realm to prevent or restore hearing, essential questions remain to be answered in both mouse models of deafness and nonhuman primates [61].

The translation from animal models into gene therapies for humans with genetic deafness has not yet begun. Nevertheless, in other systems, there are numerous (1800) clinical trials that have been either completed, are in progress, or approved regarding gene therapy [63]. Another important question is the timing of gene therapy or its age limitation. In AR hearing disorders, there could be a “point of no return”, so it should be started in the first weeks of life [64].

There are still numerous unanswered questions regarding gene therapy for hearing loss in humans. Still, despite that, the field of inner ear gene therapy is moving toward the goal of developing a powerful therapeutic tool for patients with genetic hearing loss [65].

## 5. Benefits of Early Genetic Diagnostics of Hearing Loss

**Figure 3:**
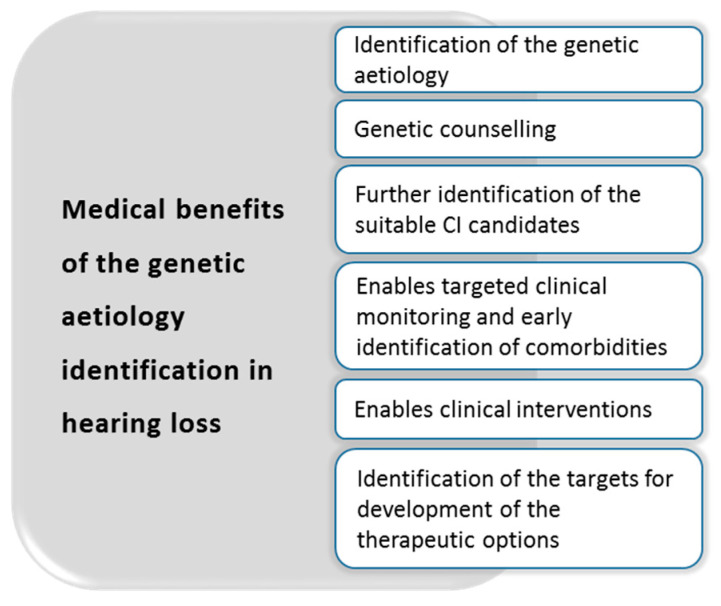
Medical benefits of the identification of the genetic etiology in hearing loss patients.

When a genetic basis of hearing loss is established, genetic counselling as part of the extended clinical genetics evaluation is offered to patients and their families [3]. There are several reasons why genetic diagnostics should be implemented early in each child with an unknown cause of hearing loss, as summarized in Figure 3.

Although CI provides a beneficial outcome for most of the hearing loss/deafness cases, factors affecting the results of CI vary among patients. The heterogeneous cause of hearing loss is thought to be one of the reasons for such variations [53]. Good CI outcomes are expected when the cause of deafness is located in the intracochlear etiology, and the deafness-affected gene is expressed inside the cochlea [53]. Children with Usher syndrome type I that are expected to develop loss of vision are advised to have CI, because of the difficulties with using sing language when blind [36].

Patients with specific hearing loss-related syndromes develop other comorbidities later during childhood or life. It is crucial to make the etiological diagnosis early to enable regular monitoring of the function of other organs [36]. That would also allow parents to prepare for the upcoming condition, and more importantly, it enables early clinical interventions when possible. Examples are monitoring of renal function in Alport syndrome, goiter, and hypothyroidism in Pendred syndrome and visual loss in Usher syndrome [54].

Furthermore, genetic testing enables other clinical interventions. The examples are early treatment such as dietary supplementation with S-adenosylmethionine in PRPS1 deficiency and treatment for hypocalcemia in hypoparathyroidism, deafness, and renal dysplasia (HDR) syndrome [54]. Furthermore, appropriate aversive/protective management is possible, such as avoidance of aminoglycoside antibiotics in patients with a m.1555A>G mitochondrial variant, avoidance of head injury in patients with EVA, avoidance of diving unattended in patients with vestibular failure, avoidance of specific medications in patients that have syndromes with long QT intervals (Jervell and Lange-Nielsen syndrome) [54].

Genetic counselling helps family members in dealing and accepting the diagnosis and its consequences. It is crucial in explaining the genetic risk for relatives and future offspring [52]. Unlike *de novo* variants, inherited variants carry a high recurrence risk and that enables options for prenatal diagnosis. It is also vital to understand the limits of genetic testing. When the result of extensive genetic testing is negative, we have to keep in mind that hearing loss could still have genetic etiology, since the disease-causing variant may be located in the region that has not been tested [52].

## 6. Conclusions

Hearing loss is one of the commonest sensory deficits. In around 60% of cases, congenital hearing loss has genetic etiology. The progress of medicine has made early genetically diagnostic of children with hearing loss possible. It is essential to make an early etiological diagnosis of hearing loss as it can have numerous significant consequences on the affected child’s and its family’s health and quality of life. Nevertheless, in numerous cases, the prediction of the phenotypic outcome based on the genetic background is difficult and imprecise and therefore, merits additional studies. 

## Figures and Tables

**Table 1:** Gene panel for next generation sequencing based on the Deafness Variation Database (http://deafnessvariationdatabase.org/) [13] used at the Clinical institute for Special Laboratory Diagnostics at the University Children’s Hospital, University Clinical Centre Ljubljana, Slovenia.

*ACTG1*	*ADGRV1*	*AIFM1*	*ALMS1*	*ATP2B2*	*ATP6V1B1*
*BSND*	*CACNA1D*	*CCDC50*	*CDH23*	*CEACAM16*	*CIB2*
*CISD2*	*CLDN14*	*CLRN1*	*COCH*	*COL11A1*	*COL11A2*
*COL2A1*	*COL4A3*	*COL4A4*	*COL4A5*	*COL4A6*	*COL9A1*
*COL9A2*	*CRYM*	*DCDC2*	*DFNA5*	*DIABLO*	*DIAPH1*
*DIAPH3*	*DSPP*	*EDN3*	*EDNRB*	*ESPN*	*ESRRB*
*EYA1*	*EYA4*	*FGF3*	*FGFR1*	*FGFR2*	*FOXI1*
*GATA3*	*GIPC3*	*GJB2*	*GJB3*	*GJB6*	*GPSM2*
*GRHL2*	*GRXCR1*	*GSDME*	*HARS2*	*HGF*	*HOMER2*
*HSD17B4*	*ILDR1*	*KARS*	*KCNE1*	*KCNJ10*	*KCNQ1*
*KCNQ4*	*KITLG*	*LARS2*	*LHFPL5*	*LOXHD1*	*LRTOMT*
*MARVELD2*	*MET*	*MIR96*	*MITF*	*MSRB3*	*MYH14*
*MYH9*	*MYO15A*	*MYO3A*	*MYO6*	*MYO7A*	*NARS2*
*NLRP3*	*OPA1*	*OTOA*	*OTOF*	*PAX3*	*PCDH15*
*PDZD7*	*PEX1*	*PEX6*	*PJVK*	*POLR1C*	*POLR1D*
*POU3F4*	*POU4F3*	*PRPS1*	*PTPRQ*	*RDX*	*SERPINB6*
*SIX1*	*SIX5*	*SLC17A8*	*SLC22A4*	*SLC26A4*	*SLC26A5*
*SMPX*	*SNAI2*	*SOX10*	*STRC*	*TBC1D24*	*TBX1*
*TCOF1*	*TECTA*	*TIMM8A*	*TJP2*	*TMC1*	*TMEM132E*
*TMIE*	*TMPRSS3*	*TNC*	*TPRN*	*TRIOBP*	*TWNK*
*USH1C*	*USH1G*	*USH2A*	*WFS1*	*WHRN*	
